# Higher Physical Activity Is Associated with Increased Attentional Network Connectivity in the Healthy Elderly

**DOI:** 10.3389/fnagi.2016.00198

**Published:** 2016-08-22

**Authors:** Geon Ha Kim, Kiho Im, Hunki Kwon, Sang Won Seo, Byoung Seok Ye, Hanna Cho, Young Noh, Jong Min Lee, Sung Tae Kim, Sang Eon Park, Hojeong Kim, Jung Won Hwang, Sue J. Kang, Jee Hyang Jeong, Duk L. Na

**Affiliations:** ^1^Department of Neurology, Ewha Womans University Mokdong Hospital, Ewha Womans University School of MedicineSeoul, Korea; ^2^Department of Neurology, Samsung Medical Center, Sungkyunkwan University School of MedicineSeoul, Korea; ^3^Ewha Brain Institute, Ewha Womans UniversitySeoul, Korea; ^4^Division of Newborn Medicine, Boston Children's Hospital, Harvard Medical SchoolBoston, MA, USA; ^5^Department of Biomedical Engineering, Hanyang UniversitySeoul, Korea; ^6^Neuroscience Center, Samsung Medical CenterSeoul, Korea; ^7^Department of Clinical Research Design and Evaluation, SAIHST, Sungkyunkwan UniversitySeoul, Korea; ^8^Department of Health Sciences and Technology, SAIHST, Sungkyunkwan UniversitySeoul, Korea; ^9^Department of Neurology, Yonsei University College of MedicineSeoul, Korea; ^10^Department of Neurology, Gangnam Severance Hospital, Yonsei University College of MedicineSeoul, Korea; ^11^Department of Neurology, Gachon University Gil Medical CenterIncheon, Korea; ^12^Department of Radiology, Samsung Medical Center, Sungkyunkwan University School of MedicineSeoul, Korea; ^13^College of Nursing, The Research Institute of Nursing Science, Seoul National UniversitySeoul, Korea

**Keywords:** physical activity, brain network, attention, graph analysis, healthy elderly

## Abstract

The purpose of this study was to demonstrate the potential alterations in structural network properties related to physical activity (PA) in healthy elderly. We recruited 76 elderly individuals with normal cognition from Samsung Medical Center in Seoul, Korea. All participants underwent the Cambridge Neuropsychological Test Automated Battery and 3.0T brain magnetic resonance imaging (MRI). Participants were subdivided into quartiles according to the International Physical Activity Questionnaire scores, which represents the amount of PA. Through graph theory based analyses, we compared global and local network topologies according to PA quartile. The higher PA group demonstrated better performance in speed processing compared to the lower PA group. Regional nodal strength also significantly increased in the higher PA group, which involved the bilateral middle frontal, bilateral inferior parietal, right medial orbitofrontal, right superior, and middle temporal gyri. These results were further replicated when the highest and the lowest quartile groups were compared in terms of regional nodal strengths and local efficiency. Our findings that the regional nodal strengths associated with the attentional network were increased in the higher PA group suggest the preventive effects of PA on age-related cognitive decline, especially in attention.

## Introduction

As the population aged 60 years or older is rapidly increasing, their number is expected to more than triple by 2100, increasing from 784 million in 2011 to 2 billion in 2050 and 2.8 billion in 2100 (United Nations, 2011). Therefore, age-related cognitive decline and development of dementia have been one of the most pressing health issues (Wimo et al., 2010). Unfortunately, pharmaceutical trials have been unsuccessful at preventing or treating dementia, which has prompted investigations to identify non-pharmacological strategies to prevent dementia, such as lifestyle adjustment to reduce modifiable risk factors, including smoking, hypertension, and diabetes (Middleton and Yaffe, 2009, 2010).

In numerous studies, the value of life style modification has been addressed as a strategy for prevention of dementia (Benedict et al., 2013; Blumenthal et al., 2013). In particular, physical activity (PA) is considered one of the most important components assumed to contribute to healthy aging. Many neuroimaging studies have supported the positive effects of PA on brain structure and function. One cross-sectional study showed a positive correlation between amount of PA and gray matter volumes in the prefrontal and cingulate cortices (Floel et al., 2010). A recent interventional study also revealed that the level of PA was positively associated with memory scores and gray matter volumes in the prefrontal and cingulate cortices (Ruscheweyh et al., 2011).

A myriad of evidence has suggested that a healthy human's brain is a large scale network with “small-world” topology (Hagmann et al., 2007, 2008; Gong et al., 2009), which is characterized by an optimal balance between the integration and segregation of information for efficient use (Latora and Marchiori, 2001). Considering that the organization of brain networks is affected in various degenerative diseases, crucial evidence for dementia prevention could be provided by determining whether the amount of PA positively influences the structural brain network in the elderly, which, however, is not well-established.

The purpose of this study was, therefore, to compare the structural brain network properties and cognitive functions in the healthy elderly according to level of PA in daily life.

## Methods

### Participants

We recruited 90 participants aged 60 years or older from Samsung Medical Center between March and August 2011. All participants met the following criteria: (1) Korean version of the Mini Mental Status Examination (K-MMSE; Han et al., 2008) scores ≥ 26; (2) years of education ≥ 6; (3) no known history of dementia or significant cognitive impairment; (4) no visual or hearing impairment severe enough to interfere with questionnaire response; (5) no history of major neurological or psychiatric illnesses; (6) no history of medication that could affect cognitive function; and (7) no major medical problems. To minimize the influence of subclinical degenerative conditions, three participants who scored two standard deviations (*SD*) or more below the population norm on tests of immediate learning and/or delayed recall scores of the Seoul Verbal Learning Tests (Kang and Na, 2003) were excluded. Of the remaining 87 participants, four refused to undergo magnetic resonance imaging (MRI). We further evaluated the 83 people who agreed to MR imaging, in order to exclude participants with territory infarctions, hemorrhage, or high signal abnormalities on MRI associated with etiologies other than mild ischemia, such as radiation injury, multiple sclerosis, vasculitis, or leukodystrophy. We only included patients with mild ischemia, which was defined as a cap or band ≤5 mm and a deep white matter lesion <10 mm. Two of the 83 participants were excluded due to structural abnormalities on MRI. Five others were excluded because they did not complete the questionnaire regarding PA. Therefore, the final 76 participants were further subdivided by quartile according to multiples of the resting metabolic rate (MET)-hour/week values.

Written informed consent was obtained from all participants and the study protocol was approved by the Institutional Review Board of Samsung Medical Center.

### Amount of physical activity (PA)

The amount of PA was assessed using the International Physical Activity Questionnaire (IPAQ; Chun, 2012). The total PA of each individual was measured as MET-hour/week, which was calculated by multiplying of the MET-hour scores by the number of performance days per week. The participants were divided by quartile according to their amount of PA (Table 1).

**Table 1:** **Demographic characteristics of the study participants**.

	**Quartile of physical activity (MET-h/week)**				
	**Quartile 1 (<21.6) (*n* = 19)**	**Quartile 2 (21.6−46.3) (*n* = 19)**	**Quartile 3 (46.4~81.3) (*n* = 19)**	**Quartile 4 (>81.3) (*n* = 19)**	***p*-value**
Age, years	66.7 ± 5.4	66.8 ± 5.5	67.3 ± 4.0	68.2 ± 5.0	0.798
Sex, *n* (% female)	14 (73.7%)	16 (84.2 %)	12 (63.2%)	11 (57.9%)	0.298
Education, years	13.7 ± 3.0	12.9 ± 3.8	13.4 ± 3.3	14.3 ± 4.4	0.724
K-MMSE	29.3 ± 0.9	29.0 ±1.5	29.3 ± 0.7	28.8 ± 1.5	0.431
IPAQ(MET-h/week)	12.7± 5.6	34.7 ± 6.1	63.9 ± 10.3	103.3 ± 17.4	< 0.001
Diabetes, *n* (%)	0 (0%)	1 (5.3%)	1 (5.3%)	1 (5.3%)	0.806
Hypertension, *n* (%)	3 (15.8%)	8 (42.1%)	3 (15.8%)	7 (36.8%)	0.234
Hyperlipidemia, *n* (%)	2 (13.3%)	8 (42.1%)	3 (15.8%)	1 (5.3%)	0.146
Cardiac disease, *n* (%)	0 (0%)	2 (13.3%)	1 (5.3%)	2 (13.3%)	0.825
*APOE* ε4 carrier^a^, *n* (%)	2/18 (11.1%)	6/17 (35.3%)	3/17 (17.6%)	2/19 (11.8%)	0.232
Geriatric depression scale	10.8 ± 5.6	7.9 ± 6.0	9.4 ± 6.1	6.3 ± 6.3	0.130
Geriatric anxiety scale	4.2 ± 4.8	3.6 ± 4.2	5.5 ± 4.6	4.0 ± 5.8	0.641
Quality of life	86.0 ± 9.5	94.6 ± 13.5	92.5 ±14.1	93.8 ± 14.7	0.169

a *APOE analysis was performed in 69 participants; 7 individuals refused the test. K-MMSE, Korean version of the Mini Mental Status Examination*.

### Cambridge neuropsychological test automated battery (CANTAB)

We used seven tests from the CANTAB, which included the three visual memory tasks of Delayed Matching to Sample (DMS), Pattern Recognition Memory (PRM), and Paired Associates Learning (PAL); two executive function or working memory tests, namely Spatial Working Memory (SWM) and Stockings of Cambridge (SOC); and the two attention tests called Reaction Time (RTI) and Rapid Visual Information Processing (RVIP). Detailed descriptions of the tests are available on Cambridge Cognition's website (http://www.cambridgecognition.com/academic/cantabsuite/tests).

#### Delayed matching to sample (DMS)

The subject is asked to touch the pattern that matches the sample. In some trials the sample and the choice patterns are shown simultaneously, whereas in others a delay (of 0, 4, or 12 s) is introduced between sample and the choice patterns. This task measures visual memory function in a four-choice delayed recognition memory paradigm. *The percentage of correct solutions* for all delay conditions gives a good overall impression of visual memory ability. Higher score is indicative of better test results. *DMS Errors* report the number of errors. An error occurs if the subject does not select the correct box in the first response. A lower score is better.

#### Rapid visual information processing (RVP)

This is a visual continuous performance task. Subjects are instructed to detect target sequences of digits (for example, 2-4-6, 3-5-7, 4-6-8) and to register responses using the press pad. Target sequences occur at the rate of 16 every 2 min. *A*′ *(A prime)* is the signal detection measure of sensitivity to the target, regardless of response tendency (range 0.00–1.00; bad to good). In essence, this measures how efficient the subject is at detecting and following target sequences.

#### Pattern recognition memory (PRM)

This tests visual recognition memory in a two-choice forced discrimination paradigm. A white box appears in the center of the computer screen, inside which digits, from 2 to 9, appear in a pseudo-random order at the rate of 100 digits per minute. The test is divided into two parts; a “warm-up” practice phase which lasts for 2 min (slow mode, 5 min) and is not scored, and a test phase which lasts for 4, 6, or 10 min, depending on the test mode, the last three/five/seven and a half of which are assessed. The subject is presented with a series of 12 visual patterns, one at a time, displayed in the center of the screen. These patterns are designed so that they cannot easily be given verbal labels. In the recognition phase, the subject is required to choose between a pattern they have already seen and a novel pattern. In this phase, the test patterns are presented in the reverse order of the original order of presentation. *PRM Percent correct* measures the number of correct responses, expressed as a percentage, with higher values indicating better pattern of recognition memory.

#### Reaction time (RTI)

In this test, the subject's speed of response to a visual target where the stimulus is either predictable (simple reaction time) or unpredictable (choice reaction time) is evaluated. *Five-choice reaction time* is the speed at which the subject releases the press pad button in response to a stimulus in any one of five locations. A lower time is better.

#### Spatial working memory (SWM)

This task assesses the subject's ability to retain spatial information and manipulate stored items in forms of working memory. The subject must touch each box in turn until one opens with a blue token inside (a search). When a blue token has been found, the subject has to place it in the right column (“home”) by touching the right-hand side of the screen. The subject must then begin a new search for the next blue token. It may be in any of the boxes that so far have been empty. This is repeated, until a blue token has been found in every box on the current screen. Touching any box in which a blue token has already been found is an error. The subject decides the order in which the boxes are searched. The computer determines the number of empty boxes that must be visited (discounting errors). Performance at the harder levels of this task is enhanced by the use of a heuristic search strategy. *SWM Between errors* are defined as times the subject revisits a box in which a token has previously been found. Lower number of visits is better. *SWM Strategy* is obtained by counting the number of times the subject begins a new search with a different box for six- and eight-box problems only. A high score represents poor use of this strategy and a low score equates to effective use of strategy.

#### Stockings of cambridge (SOC)

The task is similar to the “Tower of London” test and assesses the subject's ability to engage in spatial problem solving. The subject must use the balls in the lower display to copy the pattern shown in the upper display. The balls may be moved one at a time by touching the required ball, then touching the position to which it should be moved. The time taken to complete the pattern and the number of moves required are taken as measures of the subject's planning ability. This test requires substantial demands on executive function. “*Problems solved in a minimum number of moves”* is the fundamental measure, and records the number of occasions upon which the subject successfully completes a test problem in the minimum possible number of moves. A higher score is better.

#### Paired associate learning (PAL)

This test assesses simple visual pattern and visuospatial associative learning, which contains aspects of both delayed response procedure and a conditional learning task. For each stage, boxes are displayed on the screen. All are opened in a randomized order. One or more of them will contain a pattern. The patterns shown in the boxes are then displayed in the middle of the screen, one at a time, and the subject must touch the box where the pattern was originally located. Each stage may have up to 10 attempts (trials) in total (the first presentation of all the shapes, then up to 9 repeat presentations). If the subject makes an error, the patterns are re-presented to remind the subject of their locations. When the subject gets all the locations correct, the test proceeds to the next stage. If the subject cannot complete a stage correctly, the test terminates. *PAL Total errors* reports the total number of errors. Lower is better.

### Depression, anxiety, and quality of life

Participants were assessed for depression and anxiety using the Korean version of the Geriatric Depression Scale (Bae and Cho, 2004) and the Korean version of the Geriatric Anxiety Inventory (Kim et al., 2014), respectively. Quality of Life was also evaluated with the World Health Organization Quality of Life (WHOQOL-BREF) Scale (Min et al., 2002).

### MRI acquisition

All participants underwent brain MRI using the same imaging protocols and the same MRI scanner (Achieva, Philips 3.0T, Eindhoven, Netherlands) employing the following six different techniques: 3D T1 TFE, FLAIR, T1 REF, T2, FFE, and DTI. The 3D T1 turbo field echo (TFE) MR images were acquired with the following imaging parameters: sagittal slice thickness, 1.0 mm; no gap; repetition time (TR), 9.9 ms; echo time (TE), 4.6 ms; flip angle, 8°; and a matrix size of 480 × 480 pixels. The second technique was fluid-attenuated inversion recovery (FLAIR) MR images, the images of which were acquired with an axial slice thickness of 2 mm; no gap; TR of 11000.0 ms; TE of 125.0 ms; a flip angle of 90°; and a matrix size of 512 × 512 pixels. The third technique was T1 reference (REF) MR imaging; images were obtained with an axial slice thickness of 4 mm; no gap; TR of 545 ms; TE of 10 ms; a flip angle of 70°; and a matrix size of 512 × 512 pixels. T2 MR images were acquired using an axial slice thickness of 5.0 mm; inter-slice thickness of 1.5 mm; TR of 3000.0 ms; TE of 80.0 ms; a flip angle of 90°; and a matrix size of 512 × 512 pixels. T2 fast field echo (FFE) images were obtained using the following parameters: axial slice thickness, 5.0 mm; inter-slice thickness, 2 mm; TR, 669 ms; TE 16 ms; a flip angle, 18°, and a matrix size of 560 × 560 pixels. All axial sections were obtained parallel to the anterior commissure-posterior commissure line. The diffusion tensor images (DTI) were acquired by diffusion-weighted singleshot echo-planar imaging with specific parameters: TE, 60 ms; TR, 7696 ms; a flip angle, 90°; b-factor, 600 s/mm^2^; matrix dimensions, 128 × 128; 70 axial sections. With the baseline image without weighting, diffusion-weighted images were acquired from 45 different directions.

### Graph theoretical analysis of brain networks

Recent advances in graph theoretical analysis methods have enabled the modeling of a human brain as a large-scale network representing a collection of nodes and the lines (edges) connecting the two nodes (Sporns et al., 2005). Nodes and edges, the two basic elements of a brain network, represent brain regions and functional, or anatomical connectivity, respectively (Rubinov and Sporns, 2010).

In a large-scale brain network, network *integration* refers to the ability to rapidly combine specialized information from distributed nodes, which can be quantified into measures, such as shortest path length and global efficiency. Network *segregation*, represents specialized processing within densely interconnected groups of nodes, which can be divided into measures of clustering coefficienct and transitivity (Rubinov and Sporns, 2010). Anatomical and effective networks are simultaneously highly segregated and integrated, and consequently have small-world topologies (Rubinov and Sporns, 2010).

In this study, graph theoretical analyses were carried out on weighted connectivity networks using the Brain Connectivity Toolbox (http://www.brain-connectivity-toolbox.net; Rubinov and Sporns, 2010). All of the graph measures were scaled against the mean values of each graph measure obtained from 1000 matched random graphs that preserved the same number of nodes, edges, and degree sequence (Maslov and Sneppen, 2002).

### Structural connectivity network construction

#### Network node definition

We used the automated anatomical labeling (AAL) template (Tzourio-Mazoyer et al., 2002) to parcellate the entire cerebral cortex into 78 areas (39 regions in each hemisphere), which were defined as the nodes of the brain. Individual T1-weighted images were nonlinearly registered to the ICBM152 T1 template (Collins et al., 1994) in the Montreal Neurological Institute (MNI) space. The AAL atlas was transformed from MNI space to the T1 native space using inverse transformation with a nearest-neighbor interpolation method.

#### Network edge definition

Distortions in the diffusion tensor images caused by eddy currents and simple head motions were corrected by the diffusion toolbox of the FSL package (http://www.fmrib.ox.ac.uk/fsl/fdt). Diffusion tensor models were estimated and the fractional anisotrophy (FA) and the apparent diffusecoefficient (ADC) were calculated at each voxel. We reconstructed whole-brain white matter fiber tracts in the native diffusion space for each participant using the fiber assignment according to the continuous tracking algorithm (Mori et al., 1999), embedded in the Diffusion Toolkit (http://www.trackvis.org/; Wang et al., 2007). We terminated tracking when the angle between the two consecutive orientation vectors was greater than the given threshold of 45° or when both ends of the fibers extended outside of the white matter mask generated by a process of tissue segmentation.

#### Network construction

T1-weighted images were co-registered to B0 images in DTI space using linear registration. Reconstructed whole-brain fiber tracts were inversely transformed into T1 space, and the fiber tracts and AAL-based parcellated regions were also located in the same space. Two nodes (regions) were considered structurally connected by an edge when at least the end points of three fiber tracts were located in these two regions. A threshold of the number of fiber tracts was selected to reduce the risk of false-positive connections due to noise or the limitations of deterministic tractography (Lo et al., 2010; Shu et al., 2011). The number of fiber tracts and FA were calculated for a weight of each edge between two nodes. The fiber number that was determined by streamline tracking might reflect the white matter structure (Houenou et al., 2007), and has been used previously as a weight for network edges (Shu et al., 2011; Yan et al., 2011; Zhang et al., 2011; Batalle et al., 2012). The FA value is an important index to evaluate fiber integrity (Basser and Pierpaoli, 1996; Beaulieu, 2002) and is highly correlated with conductivity (Tuch et al., 2001). In this study, the value obtained by multiplying the fiber number by the mean FA along all the fibers connecting a pair of regions was used to weight the edge. Finally, weighted structural networks represented by symmetric 78 × 78 matrices were constructed for each individual.

### Network analysis

#### Nodal strength

We first computed the most fundamental and basic network measure, nodal strength. Nodal strength is defined as the sum of all neighboring edge weights of a node and is a measure of the local quantity of a network. The global mean strength of the network was also assessed by determining the average of the local strength of each node within the network.

#### Path length

To measure network integration, we calculated the average shortest path length between all pairs of nodes in the network. This is known as the characteristic path length (L), of the network (Watts and Strogatz, 1998).

#### Global efficiency

We also determined global efficiency as a measure of network integration. We measured the shortest path lengths between all pairs of nodes in the network and computed the global efficiency as the average inverse shortest path length (Latora and Marchiori, 2001).

#### Clustering coefficient

To measure network segregation, the weighted clustering coefficient of a node was computed and was defined as the likelihood of the neighborhoods being connected with each other (Onnela et al., 2005). We measured the mean clustering coefficient of a network as the average of the clustering coefficients across all nodes.

#### Transitivity

Transitivity indicates the connectedness of the nodes neighbors (if A connects to B and C, what is the likelihood of B and C being connected).

### Statistical analyses

For the baseline demographic data, an analysis of variance (ANOVA) was used for the continuous variables and the Chi-square test was conducted for the dichotomous variables. The differences in global and regional network topology by quartile of PA were also assessed with ANOVA. Linear contrast analysis was used to obtain the *p*-value for trend and the effect size of PA on brain network was estimated by partial eta-squared.

All analyses were performed using PASW 18 (SPSS Inc., Chicago, IL, USA). Significance was defined as *p* < 0.05.

Using Matlab 7.11 for Windows (Math Works, Natick, MA, USA), the regional nodal strength and local efficiency were compared only between the high and very low groups in order to maximize the effects of PA on brain networks. Statistical significance was assessed using a permutation-based test with a threshold of uncorrected *p* < 0.05 (Nichols and Holmes, 2002; Hurtz et al., 2014).

## Results

### Demographic data

No significant differences were seen across the four quartile groups in terms of age, sex, years of education, or K-MMSE score. The scores for depression, anxiety and quality of life also did not differ between the four quartile groups. The percentage of *APOE* ε4 carriers in the Quartile 2 group was higher, but did not reach statistical significance (*p* = 0.232). There was no difference in the prevalence of vascular risk factors, including diabetes, hypertension, hyperlipidemia, and cardiac disease, between the four groups (Table 1).

### Cognitive functions according to the quartile of PA

There was a significant linear trend in reaction time (RTI) according to the amount of PA, indicating that the people with a higher level of PA displayed a shorter reaction time (*p* for trend = 0.018; Table 2). No other cognitive functions, however, showed incremental trends with PA quartile.

**Table 2:** **Neuropsychological tests according to physical activity quartile**.

	**Quartile of physical activity (MET-h/week)**				
	**Quartile 1 (<21.6) (*n* = 19)**	**Quartile 2 (21.6−46.3) (*n* = 19)**	**Quartile 3 (46.4~81.3) (*n* = 19)**	**Quartile 4 (>81.3) (*n* = 19)**	***p*-value for trend**
**MEMORY**					
Delayed Matching to Sample (DMS)					
Correct response (%)	71.2 ± 14.4	71.9 ± 13.3	76.8 ± 8.9	70.2 ± 8.9	0.884
Pattern Recognition Memory (PRM)					
Correct response (%)	88.8 ± 8.8	85.9 ± 8.7	90.1 ± 9.1	86.3 ± 21.8	0.611
Paired Associate Learning (PAL)					
Total errors (adjusted)^a^	36.1 ± 30.8	30.5 ± 20.5	32.2 ± 25.9	21.1 ± 7.7	0.082
**ATTENTION**					
Rapid Visual Information Processing (RVIP)	0.9 ± 0.0	0.9 ± 0.1	0.9 ± 0.1	0.9 ± 0.0	0.611
Reaction Time (RTI)	376.7 ± 44.9	347.5 ± 64.7	352.1 ± 70.6	326.5 ± 41.9	0.018^*^
**EXECUTIVE FUNCTION**					
Spatial Working Memory (SWM)					
Between errors^a^	50.8 ± 14.6	47.8 ± 21.4	44.2 ± 20.5	53.9 ± 22.9	0.785
Strategy^a^	39.1 ± 3.8	37.1 ± 4.2	37.2 ± 3.5	38.1 ± 3.8	0.483
Stocking of Cambridge (SOC)					
Problems solved	6.8 ± 2.1	6.6 ± 1.7	7.7 ± 1.5	7.1 ± 1.4	0.298

a Lower scores represent better performance,

* *p < 0.05*.

### Structural network topology by PA quartile

#### Intracerebral volume and global network topology according to the quartile of PA

There was a linear incremental trend of intracerebral volume as a function of the amount of PA, indicating that the people with a higher level of PA displayed larger brain volumes (*p* for trend, 0.028). The effect size of PA on intracerebral volume was 0.07 (partial eta-squared, *p* = 0.146).

Among the indices that represent global topological organization of white matter networks (global nodal strength, global efficiency, path length, and clustering coefficient), only global nodal strength increased as the level of PA increased, but the correlation was not statistically significant (Table 3).

**Table 3:** **Intracerebral volume and global network topology according to physical activity quartile**.

	**Quartile of physical activity (MET-h/week)**				
	**Quartile 1 (<21.6) (*n* = 19)**	**Quartile 2 (21.6−46.3) (*n* = 19)**	**Quartile 3 (46.4~81.3) (*n* = 19)**	**Quartile 4 (>81.3) (*n* = 19)**	***p*-value for trend**
Intracerebral volume (cm^3^)	9800.1 ± 905.2	10016.0 ± 668.2	10313.8 ±770.5	10321.4 ± 870.3	0.028^*^
Global Network Topology					
Nodal strength	79.7 ± 30.9	83.9 ± 27.5	95.1 ± 24.7	87.4 ± 20.1	0.208
Global efficiency	0.9 ± 0.1	0.9 ± 0.1	0.9 ± 0.0	0.9 ± 0.1	0.954
Path length	1.5 ± 0.2	1.5 ± 0.2	1.6 ± 0.2	1.6 ± 0.2	0.175
Clustering coefficient	5.4 ± 0.9	5.2 ± 0.9	5.0 ± 0.7	5.5 ± 1.0	0.994
Transitivity	5.2 ± 1.0	5.0 ± 0.8	4.8 ± 0.7	5.5 ± 1.4	0.496

* *p < 0.05*.

#### Regional nodal strength and local efficiency according to PA quartile

Table 4 illustrates the regional nodes that showed liner incremental nodal strength; these areas included the bilateral middle frontal gyri and inferior parietal lobules, the right medial orbitofrontal gyrus, and the right superior and middle temporal gyri. In terms of local efficiency, the right superior temporal cortex displayed a significant difference according to level of PA (Table 4). The effect size of PA on the regional brain network was estimated in the right medial orbitofrontal gyrus where the linear incremental trend of regional nodal strength was most significant (*p* for trend = 0.003). The effect size measured by partial eta-squared of PA on the regional nodal strength was 0.12 (*p* = 0.03).

**Table 4:** **Brain regions showing linear trends in nodal strength and local efficiency**.

		**Quartile of physical activity (MET-h/week)**				
		**Quartile 1 (<21.6) (*n* = 19)**	**Quartile 2 (21.6−46.3) (*n* = 19)**	**Quartile 3 (46.4~81.3) (*n* = 19)**	**Quartile 4 (>81.3) (*n* = 19)**	***p*-value for trend**
Nodal strength	Left middle frontal gyrus	213.6 ± 66.3	210.7 ± 73.1	227.1 ± 76.1	272.0 ± 84.7	0.016^*^
	Left inferior parietal lobule	110.1 ± 55.3	204.9 ± 109.3	225.6 ± 122.8	190.0 ± 69.8	0.008^*^
	Right middle frontal gyrus	136.6 ± 51.2	173.9 ± 62.9	189.8 ± 69.4	174.4 ± 61.5	0.044^*^
	Right medial orbitofrontal gyrus	23.6 ± 21.5	30.0 ± 22.5	37.8 ± 22.6	46.7 ± 30.5	0.003^*^
	Right superior occipital gyrus	51.1 ± 39.5	55.5 ± 41.8	76.7 ± 44.8	75.2 ± 43.9	0.035^*^
	Right postcentral gyrus	174.6 ± 51.8	159.2 ± 65.2	185.1 ± 93.8	216.6 ± 73.0	0.045^*^
	Right inferior parietal	49.3 ± 49.3	70.3 ± 41.8	67.6 ± 41.0	95.4 ± 54.4	0.018^*^
	Right superior temporal gyrus	80.5 ± 48.1	95.0 ± 42.2	110.0 ± 56.4	116.3 ± 65.0	0.029^*^
	Right superior temporal pole	33.8 ± 24.5	39.1 ± 17.0	42.3 ± 24.4	56.1 ± 38.6	0.015^*^
	Right middle temporal gyrus	140.9 ± 72.4	165.0 ± 80.1	192.9 ± 73.2	186.2 ± 64.9	0.032^*^
Local efficiency	Right superior temporal gyrus	0.027 ± 0.025	0.028 ± 0.017	0.039 ± 0.021	0.041 ± 0.027	0.026^*^

* *P < 0.05*.

#### Topographical difference of nodal strength and local efficiency between the two extreme PA groups

**Figure 1:**
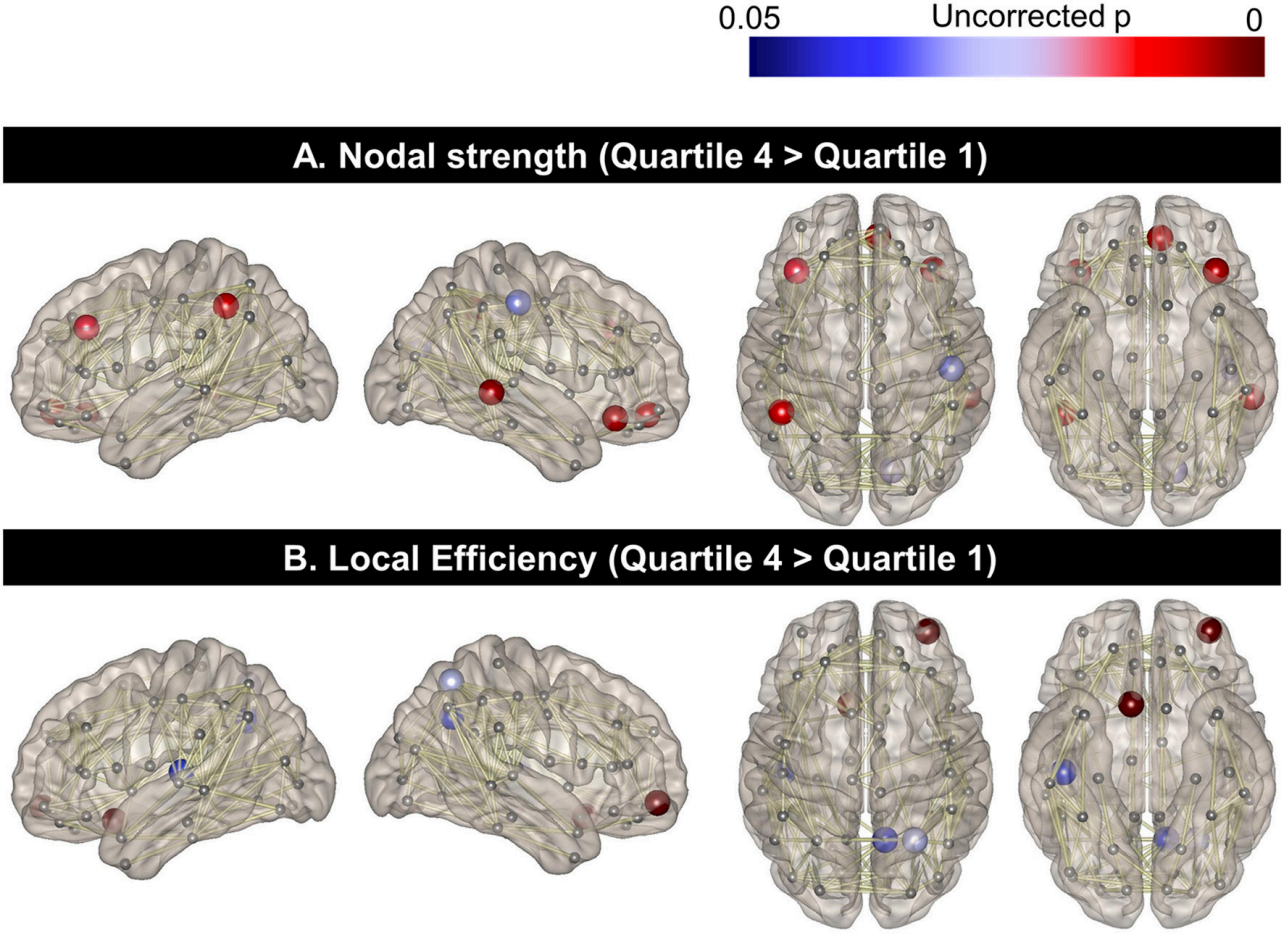
**Topographical differences in regional nodal strength and local efficiency between the two extreme PA quartiles**. Regional nodal strength in the left middle frontal cortex, orbitofrontal cortex, and inferior parietal lobules as well as the right middle temporal and orbitofrontal gyrus for Quartile 4 was greater than that of Quartile 1 **(A)**. The local efficiency of the left rectus gyrus and the right superior parietal, precuneus, and orbitofrontal cortices was also higher in Quartile 4, compared to the Quartile 1 group **(B)**.

When the regional nodal strength was compared between the two extreme groups (Quartile 1 with the lowest PA and Quartile 4 with the highest PA), the highest PA group showed greater nodal strength than the lowest PA group in the left middle frontal cortex, orbitofrontal cortex, and inferior parietal lobules as well as the right middle temporal and orbitofrontal gyrus (*p* < 0.05, uncorrected; Figure 1A). On the other hand, the highest PA group had higher local efficiency than the lowest PA group in the left rectus gyrus as well as in the right superior parietal, precuneus, and orbitofrontal cortices (Figure 1B). In contrast, there were no areas where Quartile 1 showed a higher nodal strength or local efficiency than Quartile 4.

## Discussion

This cross-sectional study allowed us to investigate whether a high level of PA in healthy elderly participants was associated with positive effects on the brain network and cognitive function, which led us to conclude that that a high level of PA was associated not only with better speed of processing, but also with increased network connectivity of attention-related brain areas.

First, we found that the elderly with a higher level of PA demonstrated better speed of processing (*p* for trend = 0.018), which was in line with previous studies showing that PA enhances cognitive reserve in the elderly (Angevaren et al., 2008; Nithianantharajah and Hannan, 2009; Carvalho et al., 2014; Frederiksen et al., 2015). The positive effects of PA on cognitive function could be explained by the fact that maintaining a higher level of PA reduces PA reduces cardiovascular risk factors such as hypertension and diabetes, which may improve cognitive function through underlying neurological mechanisms, like increasing neurotrophic factors or, reducing oxidative stress or beta-amyloid formation (Zimmerman et al., 2014).

Although previous studies have suggested that PA improves cognitive functions in the elderly, the improved cognitive domains associated with PA have been inconsistent across the studies. Several studies have indicated that a higher PA level was related to improved global cognitive function (Yaffe et al., 2001; Weuve et al., 2004), while others reported improvement only in specific cognitive domains, such as attention or cognitive speed (Angevaren et al., 2008), executive function (Eggermont et al., 2009; Frederiksen et al., 2015), or memory (Vercambre et al., 2011). As reported in prior studies as well as in the present study, cognitive improvement associated with PA or exercise was greater especially in speed of processing or tasks involving executive control than other cognitive domains (Smiley-Oyen et al., 2008; Bherer et al., 2013; Albinet et al., 2014; Dupuy et al., 2015). One account for this selective improvement could be that a higher levels of PA or exercise increase cerebral perfusion and cerebral oxygen supply to the prefrontal cortex, an area that is mainly involved in speed of processing and executive function (Brown et al., 2010; Albinet et al., 2014; Dupuy et al., 2015; Frederiksen et al., 2015).

Second, we found that the higher PA group had larger intracerebral volumes. This finding is also consistent with previous studies demonstrating that higher PA leads to increased brain reserve such as greater brain volume or neurogenesis in the elderly (Pereira et al., 2007; Nithianantharajah and Hannan, 2009). It has also been demonstrated that habitual PA is associated with greater endothelial health, which may improve angiogenesis and enhance neurogenesis (Zimmerman et al., 2014). In line with a growing body of evidence supporting that the increased brain and cognitive reserve may delay the onset of a range of brain disorders including dementia and depression (Tucker and Stern, 2011; Stern, 2012; Freret et al., 2015), our results suggest that higher PA may mitigate age-related cognitive impairment in the elderly through enhanced brain or cognitive reserve. Third, although the level of PA did not affect the global network topology of the brain in our elderly sample, the higher PA group did have greater regional nodal strength or local efficiency in areas related to attention, which included the bilateral middle frontal gyri and the inferior parietal lobules and, the right superior and middle temporal gyrus (Vossel et al., 2014; Spagna et al., 2015). It is also noteworthy that the effect size of PA on the regional nodal strength was 0.12 (partial eta-squared, *p* = 0.03), which was quite significant given that the values of 0.06 and 0.14 have been regarded as medium and large effects, respectively (Cohen, 1973; Levine and Hullett, 2002).

Furthermore, when we compared the nodal strength or local efficiency between the two extreme groups (Figure 1), the Quartile 4 group showed higher nodal strength and local efficiency in multiple cortical areas involving fronto-temporo-parietal areas, which are also known to be associated with the attentional network. Although the underlying mechanism of this selective influence of PA on the attentional network, rather than on the global network, has yet to be determined, several animal studies have suggested a relationship between aerobic exercise and increased monoamines, such as norepinephrine and dopamine (Dishman et al., 2006; Knab et al., 2009), which are important in the regulation of attention (Matthysse, 1978; Nieoullon, 2002). It is also noteworthy that the significant regions related to higher PA in our study were located relatively more frequently in the right hemisphere, which is known to play a greater role in attentional control than is the left hemisphere (Audet et al., 2000; Chica et al., 2012).

Our study had several limitations. First, the cross-sectional design of this study prevents any conclusions regarding a casual-relationship between PA and brain networks. Second, the use of MET might not reflect the exact amount of PA since we used a self-reported questionnaire to measure PA, which could be influenced by recall-bias. Third, other cognitive leisure activities of the participants should have been taken into account, as they might have also affected the attentional brain network.

Nonetheless, this study used graph analysis to reveal that a higher level of PA in daily life was associated with increased structural brain network properties in healthy elderly participants. Considering that the human attentional network involving fronto-parietal cortex is disrupted in normal aging (Andrews-Hanna et al., 2007), our results could provide crucial evidence for the preventive effects of higher PA level on age-related cognitive decline, especially in attentional skills.

## Author contributions

GH.Kim: Conception and design, data analysis and interpretation, manuscript writing. K.Im: Data analysis and interpretation, manuscript writing. JM.Lee, HK.Kwon: Data analysis and interpretation. SW.Seo, BS.Ye, ST.Kim, H.Cho, Y.Noh, SE.Park, H.Kim, JW.Hwang, SJ.Kang, JH.Jeong: Collection and assembly of data. DL.Na: Conception and design, administrative support, manuscript writing, final approval of manuscript.

## Funding

This study was supported by a grant of the Korea Health Technology R&D Project through the Korea Health Industry Development Institute (KHIDI) funded by the Ministry of Health & Welfare, Republic of Korea (HI14C3484), by the National Research Council of Science & Technology (NST) grant by the Korea government (MSIP) (No. CRC-15-07-KIER) and by Basic Science Research Program through the National Research Foundation of Korea (NRF) funded by the Ministry of Education (NRF-2015R1D1A1A01061198).

### Conflict of interest statement

The authors declare that the research was conducted in the absence of any commercial or financial relationships that could be construed as a potential conflict of interest.
